# Fat Content and Nitrite-Curing Influence the Formation of Oxidation Products and NOC-Specific DNA Adducts during *In Vitro* Digestion of Meat

**DOI:** 10.1371/journal.pone.0101122

**Published:** 2014-06-30

**Authors:** Thomas Van Hecke, Els Vossen, Julie Vanden Bussche, Katleen Raes, Lynn Vanhaecke, Stefaan De Smet

**Affiliations:** 1 Laboratory for Animal Nutrition and Animal Product Quality, Department of Animal Production, Faculty of Bioscience Engineering, Ghent University, Melle, Belgium; 2 Laboratory of Chemical Analysis, Department of Veterinary Public Health and Food Safety, Faculty of Veterinary Medicine, Ghent University, Merelbeke, Belgium; 3 Laboratory for Food Microbiology and Biotechnology, Department of Industrial Biological Sciences, Faculty of Bioscience Engineering, Ghent University – Campus Kortrijk, Kortrijk, Belgium; Utah State University, United States of America

## Abstract

The effects of fat content and nitrite-curing of pork were investigated on the formation of cytotoxic and genotoxic lipid oxidation products (malondialdehyde, 4-hydroxy-2-nonenal, volatile simple aldehydes), protein oxidation products (protein carbonyl compounds) and NOC-specific DNA adducts (O^6^-carboxy-methylguanine) during *in* *vitro* digestion. The formation of these products during digestion is suggested to be responsible for the association between red meat and processed meat consumption and colorectal cancer risk. Digestion of uncured pork to which fat was added (total fat content 5 or 20%), resulted in significantly higher lipid and protein oxidation in the mimicked duodenal and colonic fluids, compared to digestion of pork without added fat (1% fat). A higher fat content also significantly favored the formation of O^6^-carboxy-methylguanine in the colon. Nitrite-curing of meat resulted in significantly lower lipid and protein oxidation before and after digestion, while an inconsistent effect on the formation of O^6^-carboxy-methylguanine was observed. The presented results demonstrate that haem-Fe is not solely responsible for oxidation and nitrosation reactions throughout an *in* *vitro* digestion approach but its effect is promoted by a higher fat content in meat.

## Introduction

Several meta-analyses have reported a significant epidemiological association between colorectal cancer (CRC) and high intake of red and processed meat [1], [2]. However, the biochemical mechanisms underlying this association have not been completely elucidated yet. To date, the formation of cyto- and genotoxic oxidation products and genotoxic *N*-nitroso-compounds (NOCs) during digestion are considered the most plausible factors contributing to the increased risk of developing CRC when consuming large amounts of red and in particular processed meat [3]. Meat contains proteins, unsaturated lipids, haem-Fe and in case of processed meat also nitrite, which are all compounds involved in oxidation and nitrosation processes.

Major cyto- and genotoxic aldehydes such as malondialdehyde (MDA) and 4-hydroxy-2-nonenal (4-HNE), and less cytotoxic simple aldehydes, e.g. hexanal, are formed by free radical-induced damage to polyunsaturated fatty acids (PUFA). Lipid oxidation products are formed during processing, storage and preparation of meat, as well as during digestion in the gastro-intestinal tract. Next to fat, also proteins can be oxidized, resulting in the formation of protein carbonyl compounds (PCC). Lipid and protein oxidation products may cause oxidative stress and induce DNA damage due to their highly reactive nature. MDA and 4-HNE are prevailing in higher concentrations in colonic mucosae of CRC patients compared to a healthy control group [4]. Also PCC were described to be higher in plasma of CRC patients compared to a control group [5].

Previously, it was shown that volunteers on a high red meat diet had higher endogenous formation of genotoxic NOCs in feces compared to vegetarians, measured as ‘apparent total nitroso-compounds’ (ATNC) [6]. Furthermore, colonic exfoliated cells in the red meat-eating group of this study displayed higher concentrations of the NOC-specific DNA adduct O^6^-carboxy-methylguanine (O^6^-C-MeG), which were positively correlated with ATNC concentrations in feces [6]. Measurement of O^6^-C-MeG is far more specific than ATNC since the latter also include nitrosothiols and nitrosyl-heme in addition to the genotoxic NOCs.

Haem-Fe in meat has been shown to be the main promoter of the peroxidation and nitrosation pathway [3]. However, when rats were fed a diet rich in haem-Fe, the effect of haem on colonic epithelial proliferation and cytolytic activity of fecal water was lower in a low fat diet (4.2%), compared to diets containing more fat (11.5 and 20.3%) [7]. A lean beef diet (5.0% fat) high in haem-Fe did not promote colon carcinogenesis in cancer-induced rats [8]. Hence, we hypothesized that the fat content in meat might modulate the effect of haem on peroxidation and nitrosation.

Nitrite salt is widely used as a curing agent in meat products for color formation, to inhibit outgrowth of *Clostridium botulinum*, and to delay oxidative rancidity. However, nitrite-curing has also been suggested to increase NOC formation in meat products. Several studies have reported on the influence of nitrite-cured meats on the promotion of colorectal carcinogenesis. Rats consuming nitrite-cured hot-dogs (4.7% fat) had 3.7 to 5.0 fold higher NOC levels in feces compared to a control group, while consumption of beef (3.6% fat) resulted in a 2.0 to 2.9 fold increase [9]. A nitrite-cured ham diet increased cytotoxicity, lipid peroxidation and amount of ‘mucin depleted foci’ (MDF) in the colon of rats, compared to a control group [10]. However, fresh meat and processed meats differ in more properties than only nitrite-curing. Variation in haem-Fe content, fat content, and different processing procedures such as mincing and heating do not allow to draw conclusions on the isolated effect of nitrite-curing in previously mentioned studies. In a controlled study, Santarelli *et al.* [11] showed that consumption of nitrite-cured meat and oxidized meat increased ATNC and MDF in the colon of rats compared to consumption of similar non-cured and non-oxidized meat. Moreover, a human intervention study showed increased fecal ATNC when a high proportion of red meat (21.4% fat) or nitrite-cured processed meat (24.0% fat) was consumed, compared to a vegetarian diet, but no significant difference was seen between the red meat and processed meat diets [12].

Therefore, we aimed at elucidating the likely modulating effects of fat content and nitrite-curing of meat on lipid and protein oxidation markers, and the formation of NOC-specific DNA adducts during digestion. For this purpose, we used an *in* *vitro* digestion protocol adapted from Versantfoort *et al.* [13], followed by a colonic fermentation step according to Van de Wiele *et al.* [14].

## Materials and Methods

### 1. Experimental set-up

Uncured and nitrite-cured heated pork samples differing in fat content (targeted at 1, 5 or 20% fat) were compared before and after mimicked duodenal and colonic digestion. Each incubation run included all treatments in quadruplicate from which 2 duplicates underwent *in* *vitro* digestion until the duodenum and the remaining 2 until the colon. Each incubation run was performed three times with fecal inoculum originating from three different persons. In total, 2 meat samples before digestion, 6 duodenal digests and 6 colonic digests were obtained for each of the 6 prepared meat products.

### 2. Preparation of the meat samples

Commercially available lean meat samples from the *m. Longissimus dorsi* of pig were purchased in a local supermarket. The loin was manually chopped into cubes of approximately 1–2 cm^3^. Subcutaneous pork fat from one batch was added to the chopped meat to obtain a targeted total fat content of 1 (no fat added), 5 and 20%. Meat samples with added fat were first minced in a grinder equipped with a 10 mm plate, followed by grinding through a 3.5 mm plate. After grinding, nitrite-curing was applied by adding 20 g 0.6% nitrite salt/kg meat, corresponding to an added concentration of 120 mg nitrite/kg meat. All meat samples were heated in a warm water bath for 15 min after the core temperature had reached 65°C. After manufacturing, all meat samples were homogenized in three 5 s bursts using a food processor, vacuum packed and stored at −20°C until the start of the incubation.

### 3. Digestive simulations

The *in* *vitro* digestions consisted of an enzymatic digestion simulating the mouth, stomach, and duodenum, followed by a simulation of the colonic fermentation. For the enzymatic digestion, the protocol described by Versantfoort *et al*. [13] was adapted by adding oxidants and antioxidants that are normally present in digestive juices (Table 1). Hence, peroxidase [15] and NaNO_2_ [16] were added to the saliva juice, and ascorbic acid [17], H_2_O_2_ [18] and ferrous iron [18] were added to the gastric juice. Meat samples (4.5 g) were sequentially incubated for 5 minutes with 6 ml saliva, 2 hours with 12 ml gastric juice, and 2 hours with 2 ml bicarbonate buffer (1 M, pH 8.0), 12 ml duodenal juice and 6 ml bile. These enzymatic incubations were performed in quadruplicate. After completion, 2 of the 4 replicates were diluted with 44 ml H_2_O to obtain the same solid/liquid ratio as in the colon (see below), homogenized with an ultraturrax (9500 rpm) and stored at −20°C and −80°C pending analysis. The 2 remaining replicates underwent the additional colonic fermentation stage according to Van de *Wiele et al.* [14]. SHIME (simulator of the human intestinal microbial ecosystem) medium (22 ml) [19] and a human fecal inoculum (22 ml) (for preparation cfr. 2.4) were added to the digesta. The vessels were continuously flushed with N_2_ for 30 minutes to obtain an anaerobic environment. Anaerobic conditions of the flasks containing digesta at the colonic digestion stage were confirmed, using resazurin saturated test strips. Subsequently, the vessels were incubated for 72 hours while stirring at 37°C. To evaluate the rate of bacterial fermentation, total anaerobic bacteria were counted after 72 h of fermentation. One ml digestive fluid was serially diluted (10-fold) using a sterile peptone solution (1 g/l peptone, 0.4 g/l agar, 8.5 g/l NaCl and 0.5 g/l cystein), after which 0.1 ml was added on an RCM-plate (Reinforced Clostridial Medium). After 48 hours of incubation at 37°C, colony forming units (CFU) were counted and expressed as log_10_ CFU/ml digesta. As for the duodenal samples, colonic digestive fluids were homogenized by ultraturrax at 9500 rpm and subdivided in 1.3 ml aliquots while stirring on a magnetic field in dark and stored at −20°C and −80°C till analyses. Undigested control samples were obtained in duplicate by homogenizing 4.5 g meat in 82 ml H_2_O, mimicking the liquid/solid ratio in the digested samples.

**Table 1:** Composition of digestive juices used for the *in* *vitro* digestion of meat samples.

	DIGESTION^1^												FERMENTATION^7^					
	Mouth			Stomach			Duodenum						Colon					
	Saliva (1 L)			Gastric juice (1 L)			Duodenal juice (1 L)			Bile (1 L)			SHIME medium^8^ (1 L)			Cultured bacterialinoculum* (1 L)		
**Inorganic sol.**	0.90	g	KCl	2.75	g	NaCl	7.01	g	NaCl	5.26	g	NaCl	6.8	g	KH_2_PO_4_	0.51	g	NaCl
	0.20	g	KSCN	0.27	g	NaH_2_PO_4_	3.39	g	NaHCO_3_	5.79	g	NaHCO_3_	8.8	g	K_2_HPO_4_	0.01	g	KCl
	0.90	g	NaH_2_PO_4_	0.82	g	KCl	0.08	g	KH_2_PO_4_	0.38	g	KCl				0.23	g	Na_2_HPO_4_.12H_2_O
	0.57	g	NaSO_4_	0.40	g	CaCl_2_,2H_2_O	0.56	g	KCl	0.15	ml	HCl37%				0.02	g	KH_2_PO_4_
	0.30	g	NaCl	0.31	g	NH_4_Cl	0.05	g	MgCl_2_									
	1.69	g	NaHCO_3_	6.50	ml	HCl 37%	0.18	ml	HCl 37%									
**Organic sol.**	0.20	g	Urea	0.09	g	Urea	0.10	g	Urea	0.25	g	Urea	1.0	g	Arabinogalactan	0.06	g	C_2_H_3_NaO_2_S
	11.5	mg	Uric acid	0.02	g	Glucuronic acid	1.00	g	BSA	1.80	g	BSA	3.0	g	Yeast extract	33.3	g	Brain heart infusion
	25.0	mg	Mucin	0.65	g	Glucose	9.00	g	Pancreatin	30.0	g	Bile	0.4	g	Glucose	0.45	g	L-Cystein-HCl
	2.50	IU	Peroxidase^2^	0.33	g	Glucoseamine-HCl	1.50	g	Lipase				0.5	g	L-Cystein-HCl	16.0	g	Fecal matter
				17.6	mg	Ascorbic acid^4^							4.0	g	Mucin	20.0	ml	Glycerol
				1.00	g	BSA							2.0	g	Pectin			
				2.50	g	Pepsin							1.0	g	Pepton			
				3.00	g	Mucin							1.0	g	Xylan			
													4.0	g	Potato starch			
**Add**	6.90	mg	NaNO_2_^3^	10.0	µl	H_2_O_2_ (30%)^5^	0.20	g	CaCl_2_.2H_2_O	0.22	g	CaCl_2_.2H_2_O						
				11.2	mg	FeSO_4_.7H_2_O^6^												

DIGESTION^1^: based on Versantfoort *et al.* (13) unless otherwise indicated; Peroxidase^2^: Güven *et al.* (15), NaNO_2_^3^: Takahama *et al.* (16), Ascorbic acid^4^: Dabrowska-Ufniarz *et al.* (17), H_2_O_2_^5^: Nalini *et al.* (18), FeSO_4_^6^: Nalini *et al.* (18), FERMENTATION^7^: Based on Van de Wielle *et al.* (14), SHIME MEDIUM^8^: Molly *et al.* (19), *Bacterial inoculum was cultured under anaerobic conditions for 24 hours at 37°C and used immediately in the fermentation procedure.

### 4. Preparation of human fecal inoculum

Fresh fecal material was collected from 3 volunteers without known gastro-intestinal diseases and without intake of antibiotics for at least 6 months. The 3 human donors of fecal material were recruited among the laboratory personnel through informal announcement and volunteers have given their written informed consent. The research was approved by the Federal Public Service of Health, Food Chain Safety and Environment, Belgium, but was not submitted to an ethical committee for approval, nor a waiver was received. The data and volunteer information were analyzed anonymously and de-identified. All volunteers were male, meat-eaters on a Western diet, and aged 49, 26 and 38 years, respectively. Fresh fecal material was diluted in pre-cooked PBS solution (1/4; w/v), to which sodium thioglycolate (1 g/l) was added as a reducing agent. The fecal slurry was filtered by a 1 mm metal sieve to remove the particulate matter. Afterwards, the inocula were stored at −80°C on glycerol stock (20%) in different aliquots. Before use in the colonic fermentation stage, the bacterial inoculum was cultured during 24 hours at 37°C to obtain the necessary microbiotic culture. For this purpose, fecal inoculum was diluted with BHI broth (37 g/l Brain Heart Infusion and 0.5 g/l cysteine) at a 1/9 ratio. Subsequently, anaerobic conditions in the flask were reached by continuously flushing the headspace with N_2_ during 1 hour.

### 5. Chemical composition of the meat samples

The meat samples were analyzed for dry matter, crude protein and crude fat content according to the ISO 1442–1973, ISO 937–1978 and ISO 1444–1973 methods, respectively. Lipids were extracted using chloroform/methanol (2/1; v/v) [20], whereas fatty acids (FA) were analyzed as described by Raes *et al.* [21]. Briefly, FA were methylated and analyzed by gas chromatography (HP6890, Brussels, Belgium) using a CP-Sil88 column for fatty acid methyl esters (FAME; 100 m×0.25 mm×0.25 µm; Chrompack, the Netherlands). Peaks were identified, based on their retention times corresponding with standards (NuChek Prep. Inc., Sigma, Bornem, Belgium). Nonadecanoic acid (C19∶0) was used as an internal standard to quantify the individual and total FA content. The fatty acid profiles were expressed in g/100 g FAME. Residual nitrite concentrations were measured colorimetrically at 538 nm after diazotization with sulfanilamide and N-(1-naphtyl)-ethylenediamine dihydrochloride (ISO 2918–1975). Nitrite concentrations were calculated based on a standard curve obtained with SN and expressed as mg nitrite/kg meat. Hematin was determined colorimetrically by the method of Hornsey *et al*. [22], converted to haem-Fe using the formula haem-Fe = hematin *atomic weight Fe/molecular weight hematin and expressed as mg haem-Fe/100 g meat. Total Fe was determined by ICP-AES (Iris Intrepid II XSP, Thermo Electron corporation) following destruction by Bunsen burner and dry incineration at 550°C for 4 h, followed by dissolving in 3 ml concentrated HNO_3_, diluting to 10 ml bidistilled water and filtration. Total Fe was calculated based on a standard curve and expressed as mg Fe/100 g meat.

### 6. Oxidation products

MDA concentrations in digesta (−20°C) were measured by a modified method in accordance with Grotto *et al*. [23]. TBARS were formed from the reaction of MDA with 2-thiobarbituric acid in an acid environment. After extraction in 1-butanol, the absorbance of the colored complex was measured colorimetrically at 532 nm. A standard curve with 1,1,3,3-tetramethoxypropane was used and the concentration was expressed as nmol MDA/ml solution.

Levels of 4-HNE, pentanal, hexanal, heptanal, octanal and nonanal were analyzed in digesta (−80°C) through HPLC (Agilent 1200 series, provided with a degasser, autosampler, quaternary pump, column oven and fluorescence detector) using an adapted method of Holley *et al.* [24]. All solutions were purified with activated carbon and filtrated. For preparation of the cyclohexanedione (CHD) reagent, 10 g ammonium sulfate and 0.29 ml acetic acid were resolved in 100 ml, adjusted to pH 5, and purified with activated carbon. After filtration, 0.25 g CHD, dissolved in 2 ml activated carbon purified MeOH, was added to the mixture. All glassware was cleaned with ethanol and H_2_O and dried in a 100°C oven to remove contaminating aldehydes. One ml digest was mixed with 4 ml CHCl_3_:MeOH (2∶1, v/v) and 0.4 ml NaCl (9%). After 30 s vortex, the mixture was centrifuged (5 min, 1100 g) and the CHCl_3_ phase was collected. The remaining aqueous solution was mixed again with 2 ml CHCl_3_:MeOH and the CHCl_3_ phase was collected as described before. The combined CHCl_3_ phases were dried under gentle N_2_ stream. The dried residue was resolved in 0.1 ml MeOH, 0.4 ml H_2_O and 1 ml CHD reagent. After 1 h of heating at 60°C in a warm water bath, samples were filter sterilized (0.2 µm cellulose membrane filter) in dark HPLC vials. The injection volume was 80 µl and flow rate 1 ml/min. Separation was done on a Supelcosil LC-18 column (25 cm×4.6 mm, 5 µm, cat. 58295 Supelco), using stepwise elution (50% tetrahydrofuran from 0–40 min). The derivatized aldehydes were detected by a fluorescence detector at an excitation wavelength of 380 nm and an emission wavelength of 446 nm. Aldehydes were quantified using a standard curve and expressed as pmol aldehyde/ml digest.

The measurement of PCC following their covalent reaction with 2,4-dinitrophenylhydrazine (DNPH) was done according to Ganhão *et al.* [25]. This reaction leads to the formation of a stable 2,4-dinitrophenyl hydrazone product. Total carbonyl content was quantified colorimetrically at 370 nm, using a molar absorption coefficient of 21.0/(mM·cm) and expressed as nmol DNPH/mg protein. Protein concentrations were measured at 280 nm after reaction with 2 M HCl instead of DNPH, quantified using a standard curve with BSA and expressed as mg/ml solution.

### 7. O^6^-Carboxy-methylguanine and O^6^- methylguanine

The NOC-specific DNA adduct O^6^-C-MeG was quantified using U-HPLC-MS/MS [26]. In brief, 182 µl of filter sterilized sample was incubated for 18 hours at 37°C with 100 µg calf thymus (CT-)DNA. After addition of the internal standard (50 µL, 20 ng/ml O^6^-D3-MeG), the mixture was dissolved in 2 ml of 0.1 M formic acid and hydrolyzed by heating (80°C for 30 min). The hydrolysate was cooled on ice and then applied to an Oasis HLB cartridge (SPE) (30 mg, 1 ml), which was conditioned with 2 ml of 100% MeOH and equilibrated with 2 ml deionized water. After loading the hydrolysate, a vacuum suction was applied on the SPE cartridge, followed by the elution step with 2 ml of 100% MeOH. The collected fraction was evaporated to dryness (90 min, 20°C) using a SpeedVac Plus (Savant, Holbrook, NY, USA). Finally, the dried residue was dissolved in a total volume of 100 µl of mobile phase consisting of 95∶5 0.05% aqueous acetic acid:H_2_O. Chromatographic separation of the DNA-adducts was achieved by reversed phase chromatography and gradient elution. Separation of the DNA-adducts was carried out on a Nucleodur C18 ISIS column (5 µm, 250×4 mm, Machery Nagel, Düren, Germany), kept at 30°C. Analysis was performed on a triple quadrupole mass analyzer (TSQ Vantage, Thermo Electron, San Jose, USA), fitted with a heated electrospray ionization (HESI II) source operating in the positive ion mode. A standard curve of O^6^-C-MeG was made by derivatization of O^6^-carboxymethyl-deoxyguanosine (O^6^-C-MedG) with 0, 1 M formic acid at 70°C for 1 hour [27]. O^6^-C-MeG was quantified using the standard curve and the area ratio of internal standard O^6^-D3-MeG and expressed as ng O^6^-C-MeG/ml digesta.

### 8. Short chain fatty acids and NH_3_

Five ml of 10% formic acid containing internal standard (1 mg 2-ethyl butyric acid) was added to 1 ml digest. After 15 min centrifugation at 4°C and 22.000 *g*, supernatant was filtered and an aliquot was transferred into a 1.5 ml glass vial. Samples were stored at 4°C until analysis using gas chromatography (Shimadzu Corporation, ‘s-Hertogenbosch, The Netherlands) equipped with a Nukol column (30 m×0.25 mm×0.25 µm, Supelco) with a flame ionization detector. Briefly, 0.5 µl of sample was injected with the carrier gas N_2_, the injector temperature was 250°C and the inlet pressure 52.7 kPa. The temperature program was 90°C at the start of the injection, increasing 20°C/min until 160°C (kept for 8.5 min), increasing 10°C/min until 170°C (kept for 2 min). The detection temperature was 250°C. Ammoniac concentrations were measured by a colorimetrical method described by Chaney and Marbach [28].

### 9. Statistical analysis

Data were analyzed using SAS enterprise guide 5. For the data on the meat samples prior to digestion, a linear model with the fixed effects of fat content, nitrite-curing and fat content × nitrite was used. For the duodenal and colonic digestion samples, a mixed model was used with the same fixed effects and the random effect of incubation run. Data on O^6^-C-MeG were analyzed per incubation run separately due to very high variation between runs related to the use of different human fecal inocula. Tukey-adjusted *post hoc* tests were performed for all pairwise comparisons. P<0.05 was considered significant.

## Results

### 1. Meat characteristics

The characteristics of the prepared meats prior to digestion are shown in Table 2. Dry matter content and fatty acid concentrations increased, whereas protein content decreased with increasing fat content. There was an unexpected effect of nitrite and fat content × nitrite interaction for the fat content due to a lower than targeted fat content of the 20% fat uncured samples. Addition of subcutaneous pork fat to the lean meat resulted in a significantly lower proportion of LC n-3 and n-6 PUFA and higher ALA, LA and SFA proportions in the fatty acid profile of the prepared meat samples. Residual nitrite levels in the cured meat samples amounted to approximately 41%, 30% and 17% of the added amount (120 mg/kg meat), hence there was a significant effect of fat content on the residual nitrite level. Even though originating from the same pork batch, nitrite-cured meats contained significantly less Fe than the corresponding uncured meats. However, no effect was observed on haem-Fe. The pH was slightly lower in the nitrite-cured meats while no effect of fat content was observed.

**Table 2:** Composition of the pork model products used in the *in* *vitro* digestion.

Nitrite-curing		Uncured			Nitrite-cured				P-values		
Fat content (%)		1	5	20	1	5	20	RMSE	F	NC	F×NC
**Item**	**Unit**										
**Dry matter**	%	29.1^c^	31.9^b^	39.0^a^	29.3^c^	31.1^b^	43.6^a*^	0.41	**<.001**	**.002**	**<.001**
**Protein**	%	24.5^a^	21.5	17.8^b^	22.2	22.2	18.4	1.08	**.001**	.597	.171
**Fat**	%	1.6^c^	5.6^b^	17.7^a^	1.7^c^	5.4^b^	20.6^a*^	0.38	**<.001**	**.006**	**<.001**
**SFA**	g/100 g FAME	35.0^c^	38.0^b^	39.6^a^	38.3^b*^	38.3^b^	39.6^a^	0.22	**.026**	**<.001**	**<.001**
**MUFA**	g/100 g FAME	41.3	42.2	43.1	41.5	42.2	42.9	0.91	.178	.958	.942
**PUFA**	g/100 g FAME	15.4	16.0	16.4	15.4	15.9	16.1	0.23	.101	**.042**	.348
**ALA**	g/100 g FAME	0.32^b^	0.68^a^	0.79^a^	0.31^b^	0.69^a^	0.75^a^	0.02	**<.001**	.209	.868
**LC n-3 PUFA**	g/100 g FAME	0.86^a^	0.29^b^	0.22^b^	0.81^a^	0.27^b^	0.17^b^	0.02	**.002**	.058	.793
**LA**	g/100 g FAME	10.7^b^	12.9^a^	13.9^a^	11.3	12.8	13.7	0.52	**<.001**	.757	.678
**LC n-6 PUFA**	g/100 g FAME	3.84^c^	1.35^b^	0.69^a^	3.19^c*^	1.38^b^	0.69^a^	0.07	**<.001**	**.005**	**.003**
**Residual nitrite**	mg/100 g	-	-	-	4.87^c^	3.64^b^	1.98^a^	0.078	**<.001**		
**Total Fe**	mg/100 g	0.54	0.55	0.61	0.46	0.49	0.49	0.033	**.**238	**.004**	**.**495
**Haem-Fe**	mg/100 g	0.26	0.25	0.26	0.29	0.31	0.26	0.118	.627	.291	.672
**pH**	-	5.6	5.6	5.6	5.5	5.5	5.5	0.00		**<.001**	

Data (total n = 12) were analyzed using a linear model with the fixed effects of fat content (F), nitrite-curing (NC) and their interaction term (F×NC); RMSE = root mean square error; a, b, c = means for different fat content (within curing treatment) with different superscripts are significantly different (P<0.05); * = significantly different from uncured equivalent (P<0.05); SFA = saturated fatty acids; MUFA = monounsaturated fatty acids; PUFA = polyunsaturated fatty acids; ALA = α-linolenic acid (C18∶3, n-3); LC n-3 PUFA = long chain omega-3 polyunsaturated fatty acids (C20∶5, n-3; C22∶5, n-3; C22–6, n-3); LA = linoleic acid (C18∶2, n-6); LC n-6 PUFA = Long chain omega-6 polyunsaturated fatty acids (C20∶4, n-6; C22∶4, n-6; C22∶5, n-6); FAME = fatty acid methyl esters.

### 2. Total anaerobic bacterial count

Colonic digestive samples counted 7.4±0.6 log_10_ CFU/ml and was not influenced by fat content (P = .442) or nitrite-curing (P = .241) (data not shown). However, colonic digestive fluids incubated with fecal inoculum 1 contained significantly less log_10_ CFU/ml (6.8±0.4) than with inoculum 2 (7.6±0.2) and 3 (7.9±0.2).

### 3. Lipid oxidation

In the meat samples prior to digestion, MDA, pentanal and hexanal were significantly affected by fat content and nitrite-curing, the interaction term appeared also significant (Table 3). MDA was by far the most abundant aldehyde. Octanal was not detected at all, whereas 4-HNE and heptanal were detected in uncured but not in nitrite-cured meats. The concentration of nonanal was not affected by fat content, but nitrite-curing had a significant effect. MDA and volatile aldehyde concentrations were at least 2-fold up to 20-fold lower in the nitrite-cured versus the uncured meat samples. Aldehyde concentrations generally increased with increasing fat content in the uncured samples, but no clear effect of fat content on the aldehyde concentrations in the nitrite-cured samples was observed.

**Table 3:** Lipid aldehyde concentrations in uncured and nitrite-cured pork containing different amounts of fat (1, 5, 20%) before and after *in* *vitro* digestion.

Nitrite-curing		Uncured			Nitrite-cured				P-Values		
Fat content (%)		1	5	20	1	5	20	RMSE	F	NC	F×NC
	**Phase**										
**MDA**	BD	4.0^b^	5.4^a^	5.3^a^	2.0^*^	2.0^*^	1.9^*^	0.19	**<.001**	**<.001**	**<.001**
(nmol/ml)	D	9.1^b^	13.3^a^	12.6^a^	8.2^*^	8.4^*^	8.0^*^	0.51	**<.001**	**<.001**	**<.001**
	C	15.5^b^	18.0^a^	16.4^b^	14.7	14.3^*^	13.4^*^	0.85	**<.001**	**<.001**	**<.001**
**4-HNE**	BD	58^b^	221^a^	290^a^	*nd*	*nd*	*nd*	38.1	**.019**		
(pmol/ml)	D	3.2^c^	143^b^	192^a^	*nd*	11.5^b*^	77.2^a*^	31.6	**<.001**	**<.001**	**<.001**
	C	13.2^b^	28.2^a^	35.5^a^	14.1	19.5	23.3	10.6	**.005**	.079	.335
**Pentanal**	BD	87^b^	172^a^	192^a^	38	32^*^	45^*^	15.1	**.005**	**<.001**	**.007**
(pmol/ml)	D	65^b^	220^a^	211^a^	56^b^	91^ab*^	124^a*^	30.7	**<.001**	**<.001**	**<.001**
	C	73^b^	115^ab^	123^a^	75^b^	89^ab^	121^a^	25.3	**<.001**	.291	.352
**Hexanal**	BD	286^b^	1162^a^	1425^a^	75	77^*^	68^*^	155.5	**.005**	**<.001**	**.005**
(pmol/ml)	D	158^b^	581^a^	604^a^	106	154^*^	219^*^	126.3	**<.001**	**<.001**	**.002**
	C	121^b^	229^a^	252^a^	116	156	200	61.4	**<.001**	**.044**	.384
**Heptanal**	BD	18.4	18.4	24.4	*nd*	*nd*	*nd*	2.91	.202		
(pmol/ml)	D	14.5^b^	37.7^a^	27.1^x^	9.7	13.4^*^	11.3^*^	7.86	**<.001**	**<.001**	**.021**
	C	38.2	90.6	90.5	28.5^b^	74.7^ab^	105.8^a^	35.70	**<.001**	.779	.537
**Nonanal**	BD	20.2	14.9	18.6	6.5^*^	10.2	5.8^*^	2.92	.838	**<.001**	.128
(pmol/ml)	D	18.6	18.4	12.9	18.6^a^	13.1^ab^	10.4^b^	3.42	**<.001**	**.029**	.179
	C	11.7^b^	20.2^a^	24.3^a^	12.4	15.4	19.4	4.70	**<.001**	.064	.261

Malondialdehyde (MDA) was measured colorimetrically and expressed as nmol/ml digest. 4-hydroxy-nonenal (4-HNE) and the simple aldehydes were measured through HPLC-fluorescence and expressed as pmol/ml. Aldehydes in meat before digestion (BD) (total n = 12) were analyzed using a linear model with the fixed effects of fat content (F), nitrite-curing (NC) and their interaction term (F×NC). Aldehydes in duodenal (D) digests (total n = 36) and colonic (C) digests (total n = 36) were analyzed using a mixed model with the fixed effects of F, NC and F×NC and the random effect of incubation run. RMSE = root mean square error; a, b, c = means for different fat content (within curing treatment) with different superscripts are significantly different (P<0.05); * = significantly different from uncured equivalent (P<0.05); *nd* = not detected.

After digestion, MDA was by far the most abundant aldehyde, followed by hexanal. The effects of fat content, nitrite-curing and their interaction term were significant for most aldehydes. Nitrite-curing had no significant effect on pentanal and heptanal concentrations after colonic digestion. Higher concentrations of MDA were measured in both uncured and nitrite-cured meats compared to undigested meat. In contrast, concentrations of 4-HNE and volatile aldehydes were lower or similar after digestion of uncured meats, but higher for cured meats compared to before digestion. However, nitrite-cured meats had significantly lower concentrations of all measured aldehydes compared to uncured meats after the duodenal digestion and significantly lower MDA and hexanal with trends of lower 4-HNE and nonanal after colonic digestion. After duodenal digestion, nitrite-curing resulted in equal MDA independent of the fat content, while 4-HNE and pentanal were significantly higher in the cured 20% fat meat digest. Compared to their uncured equivalents, 4-HNE formation was only 2.5-fold lower in the 20% fat meat digest, while 12-fold lower concentrations were found in the 5% fat meat digest and no 4-HNE was detected in the 1% fat meat digest.

Because MDA was the only aldehyde that was present in higher concentrations in the colonic as compared to the duodenal digestion, it was hypothesized that MDA might be present in the human fecal inocula. To investigate this, blank incubations were performed without meat for the duodenal and colonic digestive simulation in a separate run. No aldehydes were detected in the blank duodenal digest, while MDA concentrations after 72 h of incubation were 5.1±0.1; 17.0±0.2 and 18.3±1.1 nmol/ml digest for the three fecal inocula respectively, indicating a significant contribution of the fecal inoculum to the MDA levels in the colonic meat digests. One might assume similar effects for the other aldehydes, but these were not measured in the present experimental set up.

### 4. Protein oxidation

Increasing PCC concentrations were observed in the following order: undigested < duodenal < colonic digests independent of nitrite-curing (Table 4). Fat content did not affect the concentration of PCC before digestion, but nitrite-cured products had lower PCC concentrations than its uncured equivalents before digestion. In the duodenal digests, uncured samples with added fat showed significantly higher PCC concentrations than the ones without added fat. Nitrite-curing led to significantly lower PCC concentrations for the duodenal digests with no significant differences between digests with different fat content. After 72 h of colonic fermentation, a marginal effect of fat content was observed with lower PCC concentrations in the 5% fat meat digests as compared to the other samples.

**Table 4:** Protein oxidation in uncured and nitrite-cured pork containing different amounts of fat (1, 5, 20%) before and after *in* *vitro* digestion.

Nitrite-curing		Uncured			Nitrite-cured				P-Values		
Fat content (%)		1	5	20	1	5	20	RMSE	F	NC	F×NC
	**Phase**										
**PCC**	BD	1.49	1.74	1.36	1.00	0.76^*^	1.28	0.261	.838	**<.001**	.**031**
(nmol DNPH/	D	1.84^b^	2.45^ab^	2.92^a^	2.02	1.91	2.22	0.566	**.007**	**.026**	.054
mg protein)	C	4.42	4.10	4.70	4.40	3.49	4.60	0.770	.**035**	.358	.601
**Protein**	BD	6.70	6.75	6.40	6.60	6.25	5.80	0.304	.227	.120	.580
(mg/ml)	D	2.10	2.15	1.91	1.87	1.83	1.56	0.327	.137	**.011**	.890
	C	1.23^b^	1.59^a^	1.23^b^	1.30^a^	1.21^ab*^	0.94^b^	0.198	**.002**	**.006**	**.022**

Protein carbonyl compounds (PCC) and protein in meat before digestion (BD) (total n = 12) were analyzed using a linear model with the fixed effects of fat content (F), nitrite-curing (NC) and their interaction term (F×NC). Aldehydes in duodenal (D) digests (total n = 36) and colonic (C) digests (total n = 36) were analyzed using a mixed model with the fixed effects of F, NC and F×NC and the random effect of incubation run. RMSE = root mean square error; a, b = means for different fat content (within curing treatment) with different superscripts are significantly different (P<0.05); * = significantly different from uncured equivalent (P<0.05).

Due to digestion, lower protein concentrations were measured in the duodenal and colonic digestive fluids compared to the meat before digestion (Table 4). Cured meats had significantly lower protein levels after duodenal and colonic simulation compared to uncured equivalent meats. From the blank incubations, it was estimated that the fecal inocula and SHIME medium contributed for 30–40% of the protein in the colonic fluid. Fecal proteins contained between 5.0 and 8.5 nmol DNPH/mg protein and hence largely contributed to the higher PCC concentrations in the meat colonic digests.

### 5. O^6^-Carboxy-methylguanine and O^6^-methylguanine

No O^6^-C-MeG was detected in meats prior to digestion or at the end of the duodenal digestive simulation (Table 5). After mimicking colonic digestion, detection was highly dependent on the applied fecal inoculum. Colonic incubation with the inoculum from volunteer 1 did not result in detectable O^6^-C-MeG. The fecal inocula from volunteers 2 and 3 resulted however in low and very high levels of O^6^-C-MeG, respectively. A significant influence of fat content was observed, with higher concentrations of O^6^-C-MeG in both cured and uncured samples containing 20% fat compared to samples lower in fat. The effect of nitrite-curing on the O^6^-C-MeG content was not consistent with significantly lower O^6^-C-MeG when using inoculum 2 and higher O^6^-C-MeG when using inoculum 3. The DNA adduct O^6^-MeG was not detected in any digestive fluid.

**Table 5:** NOC-induced DNA adduct formation by uncured and nitrite-cured pork containing different amounts of fat (1, 5, 20%) before and after *in* *vitro* digestion.

Nitrite-curing		Uncured			Nitrite-cured				P-Values		
Fat content (%)		1	5	20	1	5	20	RMSE	F	NC	F×NC
	**Phase**										
**O^6^-C-MeG**	BD	*nd*	*nd*	*nd*	*nd*	*nd*	*nd*	*-*			
(ng/ml)	D	*nd*	*nd*	*nd*	*nd*	*nd*	*nd*	*-*			
	C1	*nd*	*nd*	*nd*	*nd*	*nd*	*nd*	*-*			
	C2	46.8^c^	50.6^b^	57.8^a^	48.9^a^	44.6^b*^	50.8^a*^	0.90	**<.001**	**<.001**	**<.001**
	C3	441^b^	486^b^	697^a^	544^b^	541^b^	783^a^	55.2	**<.001**	**.043**	.824

O^6^-carboxy-methylguanine (O^6^-C-MeG) was not detected (*nd*) in meat before digestion (BD) (total n = 12) and after duodenal (D) digestion (total n = 36). O^6^-C-MeG in colonic (C1, C2, C3) digests were analyzed separately (total n = 12 per fecal inoculum), due to the very high variation between fecal inocula, using a linear model with the fixed effects of fat content (F), nitrite-curing (NC) and their interaction term (F×NC). RMSE = root mean square error; a, b, c = means for different fat content (within curing treatment) with different superscripts are significantly different (P<0.05); * = significantly different from uncured equivalent (P<0.05).

In the blank (no meat) incubations, no O^6^-C-MeG was detected when incubated with fecal inoculum 1 but high DNA adduct concentrations were measured when fecal inoculum 2 (101.0±10.6 ng/ml) and 3 (82.8±26.1 ng/ml) were applied. It should be noted that these blank incubations were performed in a separate run and are therefore not fully comparable to the test incubations.

### 6. Short chain fatty acids and NH_3_

Since the blank (no meat) incubations resulted in higher O^6^-C-MeG formation compared to the meat digests when the second fecal inoculum was used, we wondered if this might be caused by differences in the fermentation process. Therefore, we repeated 3 blank incubations and 3 digestions of uncured pork containing 5% fat, using fecal inoculum 2. There was no detection of O^6^-C-MeG in the blank or meat digests just after addition of the fecal inoculum. After 72 h of fermentation, the blank incubations contained 118.6±26.2 ng O^6^-C-MeG/ml whereas the meat digests contained only 56.5±4.9 ng O^6^-C-MeG/ml, in line with our previous findings. Significantly higher concentrations of acetate (56.7±9.0 µmol/ml vs. 33.1±0.6 µmol/ml), propionate (24.7±0.7 vs. 14.9±0.3 µmol/ml), butyrate (10.6±1.0 vs. 3.4±0.4 µmol/ml) and NH_3_ (34.2±3.1 vs. 18.7±0.8 µmol/ml) were found in the meat digests, compared to the blank digests. Repeated incubations with fecal inoculum 3 confirmed these observations (data not shown).

## Discussion

The present *in* *vitro* digestion study using pork model products allowed to investigate the effects of crucial factors that reflect the variability in composition and properties of meat products, i.e. nitrite-curing and fat content, on oxidation and nitrosation processes during digestion. By adding known pro-oxidants and antioxidants to the applied digestive juices, the *in* *vivo* situation was mimicked as close as possible. As expected, higher fat contents enhanced the formation of oxidation products and the NOC-specific DNA adduct O^6^-C-MeG during digestion. Nitrite-curing on the other hand resulted in clearly lower concentrations of oxidation products before, during and at the end of the *in* *vitro* digestion, whereas the effect on the formation of O^6^-C-MeG proved subjective for inter-individual variation. Previously, Corpet [3] suggested that the formation of oxidation products and NOCs are amongst those that could explain for the association between high red meat consumption and CRC.

In accordance to Hur *et al.* [29] who used a similar *in* *vitro* digestion model for beef patties, we observed a significant increase in MDA concentrations throughout the duodenal digestive simulation. Previously, it was reported that MDA in the small intestine is absorbed into the blood stream [30] after which it can reach several organs. Free MDA could react with DNA, resulting in the formation of the MDA-specific DNA adduct pyrimido [1,2-α] purine-10(3H)-one-2′-deoxyribose (M1dG) [31]. In contrast to MDA, highest concentrations of 4-HNE were observed in meat before digestion, after which they decreased throughout the digestion. After subtraction of the MDA values present in the applied fecal inocula, also MDA concentrations decreased from the duodenal to the colonic step. This decrease of MDA and 4-HNE during the colonic simulation could be due to reaction with proteins leading to the formation of Schiff bases or with bacterial DNA leading to DNA-adducts [31].

The antioxidative properties of nitrite were already very clear before digestion since MDA, 4-HNE, volatile simple aldehydes and PCC were clearly lower or even undetected in cured meats compared to uncured meats. Nitrite is a known antioxidant trough oxygen sequestration and may act as a precursor of the heat-stable NO-myoglobin by which the release of Fe^2+^ during heating is inhibited [32]. Consequently, less Fe^2+^ is available to catalyze the Fenton reaction, which is responsible for the initiation of oxidation processes. Since all meats were sampled fresh and oxidation in fresh meat is generally negligible, the higher lipid aldehyde and PCC concentrations in uncured meats must originate from processing (mincing, adding fat, heating). The clearly lower residual nitrite, found in the nitrite-cured meat samples when higher amounts of subcutaneous pork fat were added, could be explained by interaction of nitrite with unsaturated FA [33]. Addition of subcutaneous pork fat to meat samples resulted in an expected higher concentration of lipid aldehydes in meat, since more substrate is available for oxidation. Except for 4-HNE, the expected differences in lipid aldehydes between the 5 and 20% fat meat samples throughout digestion were not observed. Fat content of grounded beef varying between 10, 15 and 20% also had little effect on lipid oxidation parameters in the meat [34].

The lower values of oxidation products of nitrite-cured meats compared to uncured equivalents in duodenal and colonic fluids are in agreement with recently published work by Chenni *et al.* [35] who found that intake of nitrite through drinking water (1 g/l) reduced (25%) haem-induced lipid peroxidation in the colon of rats. This effect was not observed to be significant at lower doses (0.17 g/l nitrite and 0.23 g/l nitrate). Nitric oxide (NO) is able to react with ROS generating peroxynitrite, which can induce lipid peroxidation. However, NO can also react with lipophilic peroxyl radicals, generating far more stable alkyl peroxynitrates. Consequently, it was concluded that a 1∶1 ratio of NO to ROS enhances lipid peroxidation, while an excess of NO results in inhibition [36]. The reaction between nitrite and unsaturated FA leads to lower residual nitrite levels in the 20% fat meat samples and hence could modify the NO:ROS ratio and induce lipid oxidation. This could explain the higher HNE, pentanal and hexanal concentrations in the cured 20% fat meat sample compared to the cured 1 and 5% fat meat samples.

Also increased protein oxidation was observed throughout the digestion of the meat samples. The higher PCC concentrations after the colonic digestive simulation could be largely attributed to proteins in the fecal inoculum and SHIME medium. No significant differences in PCC concentrations according to fat content were noticed before digestion. It was previously described that lipid radicals may damage proteins by the binding of lipid components such as MDA and 4-HNE to the protein [37]. This could explain the higher PCC concentrations in the uncured 20% fat treatment after duodenum digestion. The lower PCC concentrations in cured meat samples before and after duodenal digestive simulations correspond with the associated lower concentrations of lipid oxidation products.

Next to higher PCC formation, we also observed a significantly higher amount of residual protein in uncured samples, indicating lower protein digestion. These findings agree with Santé-Lhoutellier *et al.* [38] who found a highly significant negative correlation between PCC concentrations and proteolytic susceptibility to pepsin. As mentioned before, MDA and 4-HNE can also react with proteins, leading to the formation of Schiff bases, which are known to play a role in protein aggregation. Previously, it was shown that a higher amount of proteins reaching the colonic fermentation, resulted in a higher formation of potentially toxic protein fermentation products such as ammonium, phenol, p-cresol and indol. However, higher formation of these products was not associated with enhanced colon cancer promotion in rats [39].

Previously, it was reported that haem-Fe is responsible for endogenous intestinal *N*-nitrosation [40]. Our results demonstrate that the fat content in meat products enhances the haem-induced intestinal *N*-nitrosation by detecting higher concentrations of the NOC-specific DNA adduct O^6^-C-MeG when a higher fat content was present in the digested meat. Salivary nitrite or nitrite-cured meats are sources for NO formation. Both NO and O_2_ preferentially diffuse in a lipid environment resulting in accumulation of both reactants in the lipid fraction to form nitrosating species. The rate of this reaction was described to be 300 times faster in a lipid compared to an aqueous environment [41], which could explain the facilitating role of fat in the formation of NOCs.

However, the effect of nitrite-curing of meat on O^6^-C-MeG formation was inconsistent in our results, being either promoting or protective depending on the applied fecal inoculum. Possibly, the effect of nitrite-curing depends on the incubation conditions (e.g. O_2_ tension), the bacterial composition of the fecal inoculum and interactions with other dietary compounds or metabolites formed during fermentation. This certainly warrants further investigation. However, the effect of nitrite-curing was perceived to be minor compared to the promoting effect of fat content for the meat products examined in the present study.

The formation of the NOC-specific DNA adduct O^6^-C-MeG was also highly dependent on the applied fecal inoculum. A difference in total anaerobic bacteria in the colonic fluids, fermentation rate or bacterial composition of the used inocula could explain the differences in the magnitude of detection. More research is required to elucidate the underlying mechanisms causing these differences.

When O^6^-C-MeG was measured in the colonic digests, blank incubations without meat displayed high values after 72 h of fermentation. Since no O^6^-C-MeG was detected just after addition of the fecal inoculum, its formation must have occurred during the fermentation. The sometimes higher values for the blank incubations than the meat digests is surprising since one expects a higher fermentation rate and more bacterial growth in the meat digests due to the higher supply of nutrients (protein, glycogen, B vitamins,…). Indeed, meat digests contained approximately 2-fold higher concentrations of NH_3_, acetate and propionate, and 3-fold higher concentrations of butyrate compared to the blank digests. However, it has been shown in rats on a high protein diet that high butyrate concentrations in the colon decreased DNA damage in the colonocytes [42]. Butyrate was also able to decrease H_2_O_2_-induced DNA damage in human colonocytes [43]. Therefore, it seems likely that the 3-fold higher butyrate production during fermentation of the meat digests interfered in the genotoxic activity of compounds. The values of O^6^-C-MeG in the blank digests can thus not be simply subtracted from the values of the meat digests.

In conclusion, our results indicate that addition of fat favors the formation of oxidation products and the NOC-specific DNA adduct O^6^-C-MeG during the digestion of pork. Nitrite-curing of meat inhibited lipid and protein oxidation but this inhibition was less pronounced throughout digestion when meat in the incubation fluids contained 20% fat. The effect of nitrite-curing on O^6^-C-MeG formation was contradictory in our results and requires more research. The measurement of O^6^-C-MeG was highly variable between fecal inocula originating from different volunteers. In this research, we simulated the passage of meat through the gastro-intestinal tract as close as possible to the *in* *vivo* situation using static *in* *vitro* incubations. Several *in* *vivo* experiments have been conducted towards this subject but these studies do not always gain insights in some mechanistic pathways as the presented *in* *vitro* work does. However, since the *in* *vivo* situation is always more complex than *in* *vitro* conditions, the outcomes in this study should be confirmed *in* *vivo*.
